# Emerging Technologies within Spine Surgery

**DOI:** 10.3390/life13102028

**Published:** 2023-10-09

**Authors:** David Foley, Pierce Hardacker, Michael McCarthy

**Affiliations:** 1 Department of Orthopaedic Surgery, Indiana University School of Medicine, Indianapolis, IN 46202, USA; 2 Indiana University School of Medicine, Indianapolis, IN 46202, USA; phardack@iu.edu; 3 Indiana Spine Group, Carmel, IN 46032, USA; mmccarthy@indianaspinegroup.com

**Keywords:** innovation, spine, surgery, technology

## Abstract

New innovations within spine surgery continue to propel the field forward. These technologies improve surgeons’ understanding of their patients and allow them to optimize treatment planning both in the operating room and clinic. Additionally, changes in the implants and surgeon practice habits continue to evolve secondary to emerging biomaterials and device design. With ongoing advancements, patients can expect enhanced preoperative decision-making, improved patient outcomes, and better intraoperative execution. Additionally, these changes may decrease many of the most common complications following spine surgery in order to reduce morbidity, mortality, and the need for reoperation. This article reviews some of these technological advancements and how they are projected to impact the field. As the field continues to advance, it is vital that practitioners remain knowledgeable of these changes in order to provide the most effective treatment possible.

## 1. Introduction

New innovations within spine surgery continue to propel the field forward. These technologies improve surgeons’ understanding of their patients and allow them to optimize treatment planning both in the operating room and clinic. Additionally, changes in the implants and procedures continue to evolve secondary to emerging biomaterials and device design. This article reviews some of these technological advancements and how they are projected to impact the field.

## 2. Methodology

Topics related to emerging technologies within spine surgery were chosen based on recent trends in the published literature, as well as popular themes seen at national and international conferences. The chosen topics included robotics, navigation, virtual reality, sagittal alignment parameters, artificial intelligence, biomaterials, motion preservation devices, and surgical training. Search queries were performed for these topics through the PubMed database. After initial screening and evaluation, additional pertinent citations were extracted from these studies’ bibliographies.

## 3. Robotics, Navigation, and Augmented Reality

Advancements in intraoperative imaging technologies have led to a subsequent evolution in the preoperative planning and intraoperative implementation of spinal surgery. Navigation, robotic assistance, and augmented reality are examples of these systems currently in use. Navigation utilizes fluoroscopy or computed tomography (CT) to generate stereotactic maps of the spine. An array sensor is attached to the patient (typically from the spinous process) and registered instruments are spatially tracked in relation to the patient’s bony anatomy. Robotics refers to the addition of a mechanical arm that locks navigated instruments into a predetermined trajectory. The potential benefits of these technologies include improved screw accuracy, better deformity correction through software planning and robotically-assisted execution, and less radiation exposure to the surgical teams [1].

Overall, the existing literature has shown at least the equivalent if not superior accuracy of navigated or robotically placed pedicle screws. Zhang et al. showed improved screw accuracy with robotics (93.4%) versus the fluoroscopic-assisted freehand technique (88.9%) [2]. Baldwin et al. similarly noted a 300% improvement in screw placement accuracy using navigation versus freehand fluoroscopy in pediatric scoliosis cases [3]. A separate review of randomized, controlled trials of thoracolumbar surgery demonstrated that robotic and navigated pedicle screw insertions in 4046 pedicles were associated with 2.66 times higher odds ratio of accurate placement and a lower risk of facet joint violations compared to fluoroscopic freehand screw placements [4]. However, a handful of studies do exist that demonstrate increased rates of malpositioning versus the freehand technique [5].

The improved accuracy of screw placement has understandably led to decreased rates of pedicle wall perforation. Existing reports cite perforation rates of 3.7–4.6% in navigated cases versus 9.5–13.4% without [6,7]. It is important to note that only a fraction of perforations lead to neurologic injury. Higher perforation risk with the use of navigation is associated with increased distance from the reference array, upper thoracic vertebrae, thin pedicles, short stature, female sex, or levels near the upper instrumented vertebrae [6].

Navigation decreases radiation exposure to the surgical team. Mendelsohn found a 2.5 times decrease in radiation using navigation versus fluoroscopically assisted surgery [8]. However, due to the need for intraoperative CT, the patient radiation exposure is significantly higher than traditional fluoroscopic methods [9]. These existing associations may be reversed with recent technological advances. McAfee et al. highlighted a radiation reduction of 80% per screw insertion with robotics versus fluoroscopic freehand insertion using modern imaging techniques [10].

Overall, the outcomes of freehand, navigated, and robotic techniques have not been shown to be significantly different. Crawford conducted a registry review of two tertiary and two community hospitals comparing one-level fusions performed for spondylolisthesis with navigated and fluoroscopically-assisted instrumentation. No differences were seen in the 90-day adverse events and 3-year revision rates between groups [11]. Similarly, Sielatychi reviewed the evidence for navigation and robotics, emphasizing that, to date, no conclusive evidence has supported that navigation has any measurable impact on patient outcomes [12]. With no major clinical difference being demonstrated, surgeons may rely on the economics of treatment to guide their choices. Dominy et al., in their registry study, demonstrated an increased charge rate for navigated versus non-navigated cervical procedures, but no statistical difference in charges for thoracolumbar procedures [13]. The use of image guidance is additionally associated with high acquisition costs and ongoing maintenance. Given these considerations, navigation and robotics, in their current state, are viewed as adjuvants to the sound surgical technique and knowledge of patient-specific anatomy, and should not be solely relied upon for the proper execution of spinal surgery.

Augmented reality (AR) and virtual reality (VR) are recent outgrowths from navigation primarily used for the visualization of deep structures during surgery. These technologies consist of three components: tracking, display, and computation systems. AR refers to virtual images that are projected over the actual physical field, while VR is an entirely virtual visual field. VR primarily uses head-mounted devices for room immersion, while AR can display on tablets, phones, or head-mounted devices. Ghaednia reviewed AR- and VR-associated technical papers with applications in spine surgery. They noted examples of these technologies being used for pedicle screw placement with an accuracy of up to 94%, with targeted cervical foraminotomy, bone biopsy, and percutaneous intervention, though often for cadaveric illustration [14,15]. VR and AR have also demonstrated value within the field of orthopedic oncology for tumor localization and within training programs for pre-surgical immersive instruction. These tools also offer significant potential for use in MIS procedures with minimal superficial cues. Concerns exist surrounding the lack of physical sensation, the initial learning curve, and headaches/dizziness/blurred vision, which may result from the use of these systems.

## 4. Sagittal Parameters

Disorders of the spine may be caused or exacerbated by atypical alignment. Consequently, understanding the parameters of spinal alignment, especially in the sagittal plane, has become an important component of operative planning. The concept of the ideal alignment is controversial and highly debated among spine surgeons. Numerous metrics are considered, including lordosis and kyphosis measures, overall spinal and local sagittal vertical axes, and the spine’s relation to the pelvis. One of the central metrics evaluated for overall sagittal spinal alignment is the sagittal vertical axis (SVA). This is measured as the distance from a C7 plumb line to the posterosuperior S1 body. Optimal values are less than 5 cm. However, clinically acceptable values for SVA likely increase with age.

**Figure 1:**
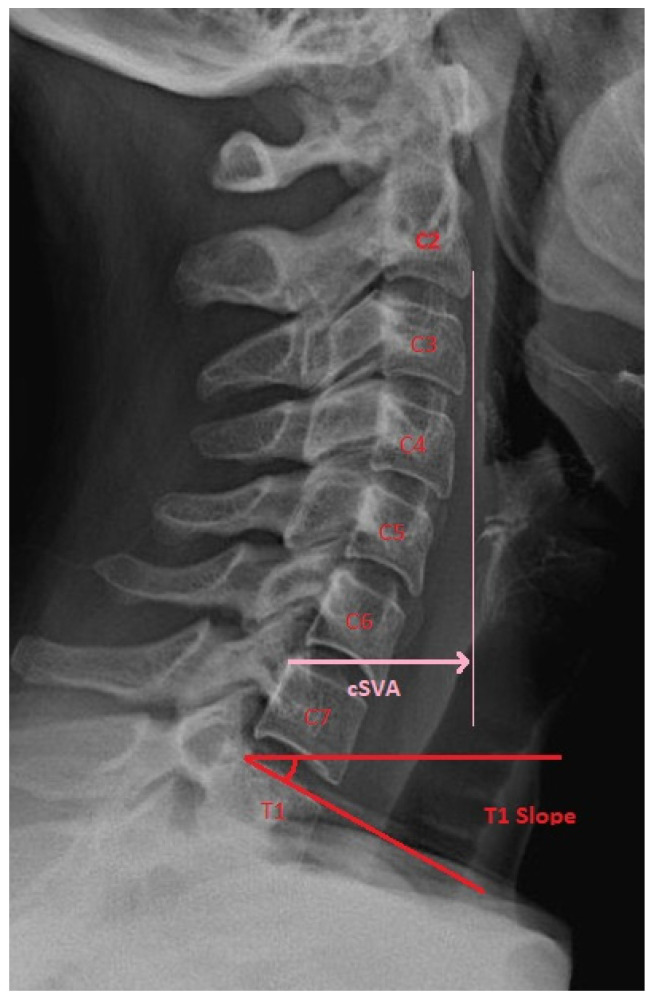
Lateral cervical spine radiograph demonstrating cervical sagittal vertical axis (cSVA) and T1 slope.

The cervical spine is considered a super-structure that is reliant on the positioning and alignment of the thoracic, lumbar, and pelvic structures. Normal cervical lordosis is 20–40 degrees. Significant effort has been put into defining the variants of cervical morphology [16]. Among the various metrics studied, the T1 slope and cervical sagittal vertical axis (cSVA) have been shown to be clinically important (Figure 1). The T1 slope is the angle between the horizontal and the superior endplate of T1. cSVA is the distance from a C2 plumb line to the posterosuperior corner of C7. Good clinical outcomes are associated with a T1 slope at or below 40° and a cSVA less than 40 mm [17]. In a large meta-analysis conducted by Azimi et al., patients with cervical pathology had increased T1 slope (healthy 24.5°, symptomatic 25.7°) and cSVA (healthy 18.7°, symptomatic 22.7°) relative to an asymptomatic control group [18]. Additionally, an elevated T1 slope and cSVA predict postoperative kyphotic deformity as the center of the head mass is anteriorly pushed [19,20]. A similar metric, the center gravity of the head to C7 SVA (CGH-C7SVA), is also associated with increased risk of postoperative kyphotic failure following laminoplasty [20]. Lastly, the occipital cervical angle (OCI) is a critical measurement in one’s ability to maintain a normal gaze with increased OCI associated with the presence and progression of ASD [21]. This measurement is the angle formed between a line drawn from the posterior border of the C4 body and McGregor’s line.

The regions of the spine are highly interdependent with a high prevalence (53%) of cervical deformity and concomitant thoracolumbar deformity [22]. Normal thoracic kyphosis (TK) and lumbar lordosis (LL) are 20–50° and 20–80°, respectively. Pelvic incidence (PI) is commonly considered when planning for surgical correction of spinal malalignment. This value is the sum of an individual’s sacral slope (the Cobb angle from the superior endplate L1 to the inferior endplate L5) and pelvic tilt (the angle formed by a line from the midpoint of the sacral endplate to the center of the femoral head and a vertical reference line). This value is considered normal when within nine degrees of lumbar lordosis. Odland et al. demonstrated that the mean PI is approximately 50° (24–69°) in asymptomatic individuals [23]. Low PI, low LL, and high pelvic tilt are associated with increased ASD incidence [24]. The correction of these parameters to within normal limits with expandable cages or hyperlordotic implants may improve patient-reported outcome measures (PROMs) [25,26,27,28].

## 5. Artificial Intelligence

Artificial intelligence (AI) refers to technologies that interpret and analyze data in ways that mimic human cognitive function. Machine learning is a subset of AI that is able to interpret huge quantities of data and makes predictions about the sampled population. This technology’s development has recently accelerated in healthcare following the development of large datasets, the availability of cloud computing, affordable high processing power computers, and open source software development [29]. Proponents claim improved patient experiences and health, healthcare-related cost reductions, and better work-life quality for providers as hopeful benefits. AI has been extensively used in general medical practice, but its utilization in spine surgery lags behind many other fields [30]. It has demonstrated value in clinical and radiographic imaging interpretation, genomic analysis, disease progression prediction, electrocardiogram pattern recognition, and numerous other unique medical applications [31].

AI’s role in automating image interpretation appears to have its largest current clinical application in spine surgery. It is able to calculate spinopelvic parameters and automatically segment vertebrae for its end users [32]. These measurements have excellent reliability and accuracy compared to expert human measurements [33,34,35,36]. Because AI is able to analyze advanced imaging on a pixel level, it can make granular assumptions about the data and modify the original image. This results in less image data input required to complete scans, decreased hardware-related distortions and motion artifacts, and MRI osseous structure analysis equivalent to that seen on CT scans [37]. Carson et al. demonstrated, in an animal model, that nerves passing through the psoas muscle may be visualized with AI for use in lateral lumbar interbody fusion. The accuracy of nerve localization in this model was greater than 95% [38].

AI has the ability to both classify imaging using existing schemes as well as form new systems based on self-directed dataset learning. It has been used to accurately grade spinal stenosis and classify intervertebral disc degeneration using MRI [39,40]. Burns et al. used machine learning to locate and classify thoracolumbar fractures based on the Denis system [41]. In one study, an unsupervised artificial neural network clustered six patient adult spinal deformity types and determined their associations with sagittal vertebral alignment and proximal junctional kyphosis [42]. Another study by Ames et al. created a four-cluster classification system for spinal deformity that predicted associated complication profiles [43].

Finding consensus for the treatment of spinal conditions is frequently difficult to obtain. A recent survey revealed that 69% of providers differed in their treatment of recurrent disc herniation [44]. AI has the potential to improve this process by guiding preoperative risk stratification and making procedure recommendations in regard to its generated outcome and complication profile [37]. Mourad et al. created a hybrid AI model that evaluated clinical symptoms, MRI, and demographic factors to determine surgical candidacy for lumbar spinal surgery. Their model performed in a similar way to a multidisciplinary team, which included five fellowship-trained spine surgeons [45]. A separate retrospective study had 86% agreement in its surgical plan, with the actual treatment course using demographics, medical history, patient-reported outcome measures, and radiographic parameters as guidance [46]. Machine learning may also be particularly useful in creating patient risk profiles, predicting clinical outcomes, and making treatment recommendations when deployed in large data registries [32].

The majority of current forecasting models have been used to predict postoperative outcomes and complications. Spine surgery-specific AI has examined satisfaction, quality of life, mJOA, mortality, hospital readmission, opioid utilization, transfusion requirements, proximal junctional kyphosis, pseudoarthrosis, infection, and other postoperative complications [43,47,48,49,50,51,52,53,54,55,56,57,58]. Khan et al. noted that newer ML-based models perform at least as well as traditional regression modeling in the prediction of postoperative outcomes following spinal cord injury [59]. Additionally, Merali demonstrated the superiority of their model over traditional statistical models for predicting outcomes following surgery for cervical myelopathy [60]. These models have shown satisfactory accuracy in a study by Scheer et al., predicting pseudoarthrosis with 91% accuracy following adult spinal deformity correction at the two-year follow-up [61].

AI has the ability to enhance spine surgery-related research. It uses multiple complex models in parallel to examine indirect relationships and generate accurate predictions. It will expand our considered measures into previously unexplored areas such as biomarkers and epigenetics [62]. However, concerns regarding the utilization of AI within spine surgery exist. Most existing studies are retrospective with curated populations examined against expert performance and do not involve real-time decisions [31]. Other concerns exist regarding bias against subgroups of samples, privacy, accidental fitting to confounders, population characteristic shifts, and the future of clinical training. Ultimately, this is a field with great potential but which remains in its infancy with low clinical conversion efficiency.

## 6. Implant Materials

Since the advent of spine surgery, practitioners have sought to find the ideal materials to promote fusion and improve alignment. High rates of complications such as implant migration and subsidence continue to motivate surgeons in this pursuit [63]. A variety of materials have been proposed as solutions to these problems, including metals and their alloys, ceramics, polymers, and composites. These materials are frequently combined in an attempt to modulate their properties in a process called “doping”. Additionally, biologic supplements such as recombinant bone morphogenic protein-2 (rh-BMP2) are used to promote the body’s innate processes toward improved fusion and healing.

### 6.1. Metals

**Figure 2:**
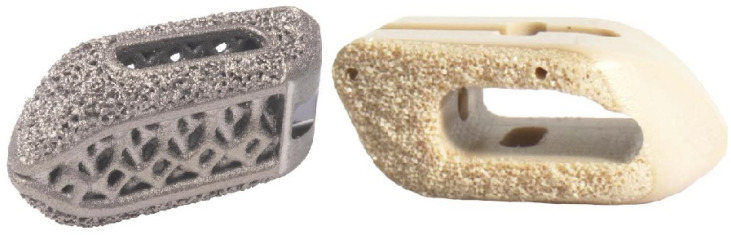
Titanium (**left**) and PEEK (**right**) lumbar interbody implants are demonstrated.

Titanium (Ti) and its alloys are widely utilized due to their biocompatibility, toughness, and fracture resistance [64]. This metal has excellent corrosion resistance and a more similar modulus of elasticity (MoE) to bone than other commonly used metals in spinal instrumentation. Adding a porous coating to the implant surface allows for osseous integration. While Ti is relatively expensive compared to stainless steel and polyetheretherketone (PEEK), it has less image artifact than many alloys. Ti implants may have, at worst, non-inferior fusion rates when compared to PEEK (Figure 2) [63,65,66]. However, multiple studies have demonstrated increased rates of subsidence in relation to PEEK and ceramics [64,67,68]. Stainless steel (FeCrNi) and cobalt-chrome (CoCr) are two other common alloys employed for spinal instrumentation. Stainless steel rods may cause pedicle stress shielding due to excessive stiffness related to its higher MoE. For this reason, PEEK and Ti rods are generally preferred. Stainless steel is notch-resistant but has substantial susceptibility to corrosion. CoCr is frequently used in scoliosis surgery as the stiffer rods maintain intraoperative correction, though with more image artifacts than Ti.

Tantalum and nitinol (50% Ti, 50% nickel) are occasionally used in spinal implants. These are more expensive than other materials such as Ti, stainless steel, and PEEK. Porous Tantalum is similar in structure to cancellous bone, has a low MoE, and a high friction coefficient, which provides excellent interface characteristics. These qualities enable load sharing, which may prevent stress shielding. Tantalum has improved pullout strength and greater osteoblast proliferation induction than Ti [69]. The current literature demonstrates in vivo animal models with early improvements in bony ingrowth versus PEEK and improved fusion rates relative to autologous bone [70]. Nitinol is a notch-sensitive metal that produces a minimal inflammatory reaction when placed in the spinal canal. It has shape memory elastic properties, which make it an optimal rod material in scoliosis corrective surgery.

### 6.2. PEEK

PEEK is the most researched biomaterial used in lumbar and cervical fusions [63]. This semi-rigid plastic is hydrophobic and unable to adhere to bone. This property may be responsible for the higher rates of implant migration and subsidence relative to Ti [71]. Proponents of its utilization point to its similar MoE as cancellous bone, reduced image artifact, and radiolucency. Much of the stress shielding seen with stiff metallic rods is reduced with PEEK rods, which allow for more axial and bending ranges of motion and anterior column load sharing [72]. Increased bending may prevent the accelerated ASD seen with stiffer metal constructs. Though this material has 2.5 times less compressive strength than Ti, PEEK interbody implants have shown better maintenance of Cobb angles than Ti cages when used for anterior cervical discectomy and fusion. PEEK has also demonstrated non-inferior clinical outcomes following spinal fusion versus Ti and ABG implants [66,72]. Overall, these three materials demonstrate similar complication profiles.

Reinforcing PEEK with carbon fiber (CFR-PEEK) or doping with Ti (TiPEEK) allows for a mixing of attributes to suit unique purposes. The use of CFR-PEEK constructs has shown potential in the field of spinal tumors. This modified plastic has excellent radiographic characteristics with no noted differences in outcomes or complications from traditional Ti or PEEK [73]. CFR-PEEK may enhance early detection of tumor recurrence and radiation dosing accuracy, though limited clinical data currently exist [74]. When PEEK is doped with Ti, it may show improved subsidence rates relative to PEEK alone. However, most studies have demonstrated similar clinical and radiographic outcomes, including ultimate fusion rates [63,66,75].

### 6.3. Hydroxyapatite

Hydroxyapatite (HA) is a phosphate mineral that may be surfaced on an implant to improve bony integration. HA-coated pedicle screws have increased pullout force and bone-to-implant contact relative to uncoated screws [76,77]. This material is frequently indicated in patients with decreased bone mineral density due to these properties. Studies have shown shorter operative times and blood loss in procedures utilizing HA versus autogenous bone graft, but no difference in final outcomes or complications [78,79].

### 6.4. rhBMP2

rhBMP2 is an alternative to an iliac crest bone graft (ICBG) applied to avoid donor site morbidity and increased wound-related complications seen with autograft harvest. rhBMP2 has superior spinal fusion rates relative to ICBG, local autograft, allograft, BMP-7, and demineralized bone graft (DMB) when used alone [63,80]. The addition of BMP2 addition to PEEK in lumbar fusion increases fusion rates and may improve PROMs relative to PEEK HA+tricalcium phosphate. It has also been shown to improve fusion rates in ACDF, but may cause potentially life-threatening prevertebral edema [65]. In rat models, rhBMP2 caused more soft tissue edema and higher inflammatory marker levels than HA-DBM [81].

### 6.5. Ceramics

Ceramics are currently being used within spinal surgery to promote fusion. Silicon nitride is a brittle, semi-radiolucent ceramic with high thermal and bacterial resistance. Because its particles are able to be resorbed, they do not contribute to particle load and subsequent osteolysis. Costs of production and subsidence rates are comparable with PEEK [68,70]. The U.S. Federal Drug Administration reported an adverse effect rate of 0.07% in human applications. Tricalcium phosphate (TCP) is another osteoinductive ceramic bone graft substitute. A randomized, controlled trial investigating ICBG versus TCP in lumbar interbody fusion showed no difference in complications or clinical outcomes, with excellent fusion rates in both treatment groups [82]. Silicon-substituted calcium phosphate is a modified form of TCP with a 93% reported radiographic fusion rate similar to that seen in rhBMP2-impregnated grafts. In one clinical study, the use of this material in interbody fusion resulted in a significant postoperative improvement in clinical outcomes [83]. Osteoinductive factors may be added to ceramics to improve their fusion rates. Stromal vascular fraction (SVF) is a component of adipose tissue that is isolated and used in conjunction with ceramics in lumbar interbody procedures. A case series evaluating TCP in lumbar interbody fusion with and without SVF showed a statistically significant improvement in fusion grade with the use of SVF at 6 months, though these differences were not maintained at subsequent follow-up [84].

### 6.6. Bioabsorbable Materials

Bioactive glass is a semi-crystalline, gel material that has antimicrobial and osteoinductive properties. Like silicon nitride, it is soluble and resorbed by the body. The remaining ions deposit Ca and Phosphorus to form hydroxyapatite. In regards to fusion, it behaves with similar efficacy to iliac crest autograft [70]. Adequate clinical studies of this biomaterial are still lacking and have yet to establish its non-inferiority [85]. Other bioabsorbable materials, such as modified polymer interbody cages and annulus fibrosis-repairing biogels, have been used with limited success [86,87]. There has been, overall, poor quality of evidence and significant bias present in most studies evaluating composite bone substitutes and factors versus autograft (ICBG) [88].

## 7. Motion Preservation

### 7.1. Cervical Disc Arthroplasty

**Figure 3:**
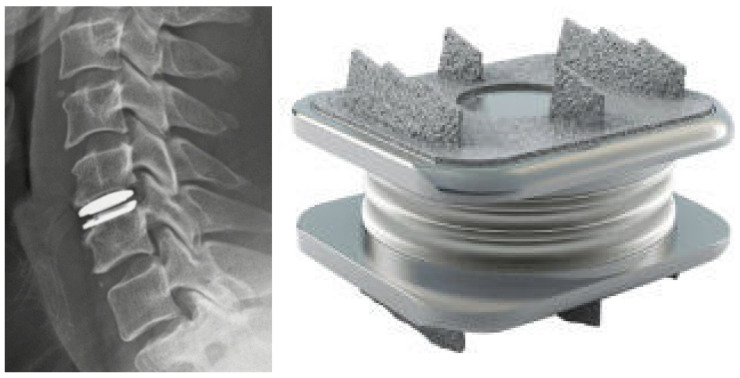
Lateral radiograph of the cervical spine following cervical disc arthroplasty at C5/6 (**left**). The M6-C^TM^ Artificial Cervical Disc (**right**).

Anterior cervical discectomy and fusion is considered the gold standard for the surgical treatment of cervical radiculopathy and is a commonly used option for cervical myelopathy. As the number of fused levels increases, pseudoarthrosis and failure rates increase [89,90]. Additionally, the loss of motion at the index level is thought to cause abnormal compensatory motion and stress transfer to the adjacent levels, leading to adjacent segment degeneration (ASD) [91]. CDA is designed to maintain ROM at the index and adjacent levels. There are currently nine FDA-approved CDA implants, with numerous others in IDE trials (Figure 3). Of these, three have received FDA approval for two-level usage. The inclusion criteria include one- or two-level cervical degenerative disc disease levels causing intractable radiculopathy or myelopathy [92]. The most common designs of arthroplasty implants consist of either a core sandwiched between two metal baseplates or a “ball-in-trough” configuration. Plastics such as ultra-high molecular weight polyethylene and elastic composites have been utilized in the core component. Early implants used metal-on-metal bearings, but this design has largely been abandoned.

Single-level CDA reliably improves neck disability index (NDI) and visual analog scale (VAS) scores. Versus ACDF, CDA results in at least non-inferior overall satisfaction and success rates, with the majority of investigations indicating its superiority [93,94,95,96,97]. CDA also demonstrates greater improvements in NDI and VAS relative to ACDF [95,96,98]. Both one-level and two-level CDA boast high rates of successful outcomes at 86% versus 96% at 1 year, respectively [99]. No difference in patient-reported outcomes measures (PROMs) has been noted between one- and two-level procedures [100,101]. However, higher overall success and satisfaction rates have been seen with two-level CDA versus two-level ACDF [92,102,103]. PROMs similarly improve in both ACDF and CDA for two-level procedures, though CDA has been demonstrated to be equivalent to superior short- and long-term improvements versus ACDF [92,100,102,104,105,106,107,108,109].

Biomechanical studies have compared ROM among single and multilevel fusion, arthroplasty, and hybrid constructs. The index level and total cervical ROM both decrease following single-level ACDF. Two-level ACDF increases adjacent-level ROM at both levels, arthroplasty has no significant change in adjacent ROM from preoperative values, and hybrid surgery increases ROM adjacent to the fused level [110,111,112]. In hybrid constructs, ROM at the arthroplasty level also increases [112]. These findings have been replicated in clinical studies with significantly less ROM at adjacent levels in hybrid constructs than in multilevel ACDF [113]. Following CDA, index-level ROM improves and is maintained at long-term follow-up [94,97,103,104,106,108,114,115,116,117]. This preservation of native ROM at the index and adjacent levels may reduce future ASD development.

Complications following CDA and ACDF include ASD, reoperation, dysphagia, and heterotopic ossification (HO). Overall, lower complication rates have been seen following CDA than following ACDF, with no significant difference in serious adverse events. Less ASD at both superior and inferior levels has been seen following arthroplasty versus fusion in one- and two-level procedures [92,95,104,116,117,118,119]. Superior adjacent-level disc height (an inversely related surrogate to ASD) decreased in both CDA and ACDF at 7 years with no significant difference between groups [120]. Jawahar et al. noted no difference in ASD between one- and two-level CDA, indicating that the presence of CDA does not affect overall ASD rates [121]. Like ASD, no difference in reoperation rates has been noted between one- and two-level CDA [101]. One- and two-level CDA demonstrated lower reoperation rates at both the index and adjacent levels versus ACDF [92,94,97,98,100,103,104,106,117,122,123]. Similarly, dysphagia following single-level CDA was demonstrated to occur at lower rates compared to ACDF at 7-year follow-up [124]. HO formation following CDA is a known potential outcome, with variable incidence rates in the literature ranging from 0% to 75% [100,108,115,125]. Its clinical significance has yet to be established [126]. Increased HO development has been seen in two-level CDA versus one-level CDA [100,101,126]. Higher rates of adjacent level ossification development (ALOD) have also been seen with ACDF than with CDA [127].

The currently available data suggest the statistically significant superiority of two-level CDA over two-level ACDF in long-term outcome measures and reoperation rates. Much of the existing data supporting multilevel CDA come from industry-funded IDE trials, with varying degrees of risk of bias [128]. Providers should consider the potential for conflicts of interest and bias when interpreting these results.

### 7.2. Laminoplasty

Laminoplasty (LP) is a procedure that, like CDA, preserves cervical ROM in an effort to reduce ASD [129]. LP has been indicated to treat myelopathy secondary to spondylosis or ossification of the posterior longitudinal ligament (OPLL). During the open door technique, an opening trough is made at the junction of the lamina and lateral mass. A second trough is made of the contralateral side with only the dorsal lamina being violated. The posterior arch is then hinged open and fixed in place using a small plate with screws, a suture, a suture anchor, and/or bone. Alternatively, the French door approach may be used with bilateral hinges and an opening through the midline. While the latter procedure potentially produces less blood loss, an open door LP is generally preferred due to greater improvements in PROMs, the maintenance of ROM, and a favorable complication profile [130]. LP is ideal for use in the lordotic spine, though the existing literature indicates it may be successful for patients with up to 13° of kyphotic alignment. Many surgeons prefer at a least neutral sagittal balance, as some loss of lordosis (approximately 5°) is expected after LP. By retaining the posterior spinous structures, the prevention of catastrophic kyphotic failure seen after laminectomy may be avoided. Most patients can expect a moderate improvement in PROMs after LP. Equal or superior PROM improvements are seen after LP versus ACDF or laminectomy [131,132,133]. An equivalent or decreased rate of complications is seen with LP than other treatments for cervical myelopathy. Posterior cervical procedures, including LP, have more wound complications, including infections, than anterior procedures. However, fewer infections are seen in LP than in a laminectomy. LP has similar postop JOA and neurological improvement, lower complications, and worse alignment correction than ACDF [134]. LP also has lower rates of C5 palsy than both ACDF and laminectomy [132,133].

## 8. Developments in Surgical Training

The foundation of spine surgery training remains hands-on, supervised learning with graduated autonomy applied in vivo. While studies have shown that this is an overall effective and safe method of education, training programs desire to provide translatable skills in consequence-free environments. Waisbrod et al. demonstrated no difference in outcomes or complications with single-level lumbar procedures performed by faculty surgeons or by trainees under supervision [135]. However, given that residents obtain varied instruction in their training and are limited by workhour regulations, innovative methodologies including synthetic and simulation models are being used to complement traditional surgical practice [136].

A key component of most curricula is bioskills training. This consists of task-oriented goals performed outside of the traditional operating room setting. Directed bioskills training improves trainee technical performance and decreases in-lab operating errors [137]. The most commonly used method of bioskills training is the utilization of human cadaveric models [138]. This established tool provides trainees with faithful exposure to anatomy and tissue [139]. Its use is limited by tissue degradation and expense concerns. Animal models also have potential benefits, particularly for minimally invasive surgical (MIS) training. Gotfryd et al. demonstrated that a swine model for MIS decompression and pedicle instrumentation training was similar and translatable to human procedures. Its value in intradiscal procedures was more limited [140]. Synthetic modeling with products, such as Sawbones, is another current practice [138,141]. It is a cheap alternative that provides haptic feedback but with significantly less tissue fidelity. Consequently, researchers are working to improve synthetic tissue biomechanical characteristics. For instance, Gragnaniello et al. developed a polymer that can be injected into animal or human cadaveric models in order to mimic spinal pathology [142]. Mixed reviews exist regarding perceptions of bioskills training efficacy. Instructing surgeons have reported that performance by their trainees in the bioskills laboratory would moderately encourage them to advance their trainees’ participation in the operating room [138].

While the utilization of surgical simulations is increasing in training programs, a deficiency in quality systems exists [141]. Examples of simulators include virtual reality, augmented reality, and synthetic models with adapted imaging systems. Furst et al. created an electromagnetic tracking system using a synthetic patient model that simulates fluoroscopic thoracolumbar pedicle screw placement [143]. These systems are valuable for practicing intraoperative tasks, such as pedicle screw instrumentation or dural repairs. Other skills, such as those specific to MIS procedures, have steep learning curves and are thus excellent candidates for pre-patient training. Simulators target surgical skill improvement but are also evaluation tools. A study by Wang et al. was able to differentiate novice, intermediate, and expert MIS operators by evaluating the task completion time, the instrument movement distance, and the number of movements [139]. Virtual reality simulators allow for full immersion and may be useful for training team-oriented tasks [144]. These systems do not provide haptic feedback, are expensive, and their effectiveness has not yet been validated.

Preoperative surgical planning software frequently accompanies robotic surgical systems. It allows surgeons to see the impact of planned interventions such as osteotomies, rod curvature, screw positioning, and various interbody implants. This may be utilized as a teaching tool for residents and fellows when developing preoperative plans, particularly in complex deformity correction. However, concerns exist regarding the use of robotics in training, leading to overreliance, lower anatomic knowledge, and less tactile skill development [145].

## 9. Conclusions

Emerging technologies within spine surgery have transformed the way that surgeons practice. Advancements allow for enhanced preoperative decision-making, improved patient outcomes, and better intraoperative execution. Changes in implant materials and device options also allow for fewer potential complications, such as subsidence, ASD, loss of alignment correction, and need for reoperation. As the field continues to advance, it is vital that practitioners remain knowledgeable of these changes in order to provide the most effective treatment possible.

## Data Availability

Data sharing not applicable.
