# Correction: Foley et al. Emerging Technologies within Spine Surgery. *Life* 2023, *13*, 2028

**DOI:** 10.3390/life14060683

**Published:** 2024-05-27

**Authors:** David Foley, Pierce Hardacker, Michael McCarthy

**Affiliations:** 1 Department of Orthopaedic Surgery, Indiana University School of Medicine, Indianapolis, IN 46202, USA; 2 Indiana University School of Medicine, Indianapolis, IN 46202, USA; phardack@iu.edu; 3 Indiana Spine Group, Carmel, IN 46032, USA; mmccarthy@indianaspinegroup.com

In the original publication [1], there was a mistake in Figure 1 as published. The plumb line drawn for the calculation of the cervical sagittal vertical axis should originate from the center of the C2 body. In the original figure, the line incorrectly begins at the anterior body. The corrected Figure 1 appears below. The authors state that the scientific conclusions are unaffected. This correction was approved by the Academic Editor. The original publication has also been updated.

## Figures and Tables

**Figure 1:**
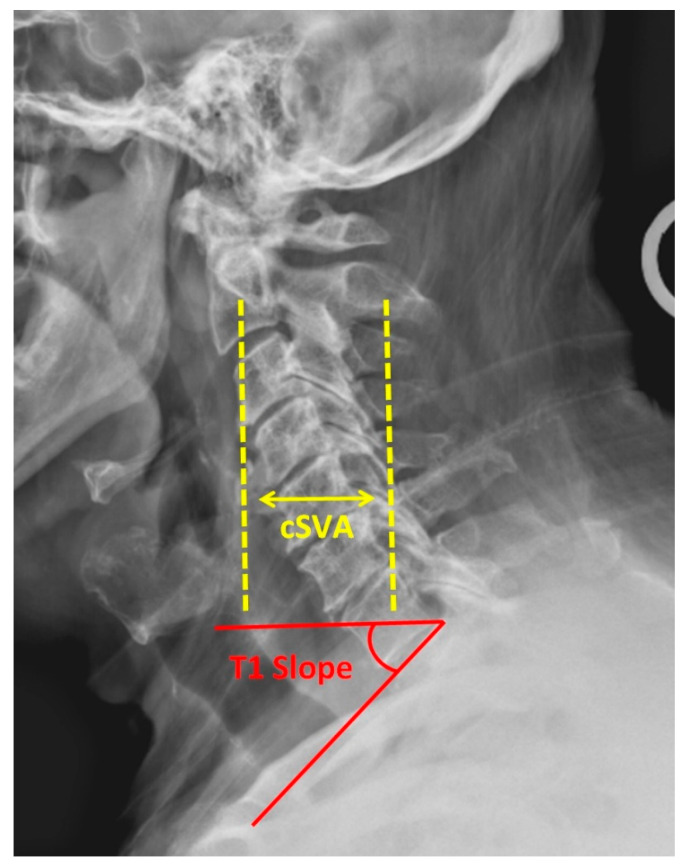
Lateral cervical spine radiograph demonstrating cervical sagittal vertical axis (cSVA) and T1 slope.
